# Emerging Insights on Brazilian Pepper Tree (*Schinus terebinthifolius*) Invasion: The Potential Role of Soil Microorganisms

**DOI:** 10.3389/fpls.2016.00712

**Published:** 2016-05-24

**Authors:** Karim Dawkins, Nwadiuto Esiobu

**Affiliations:** Microbial Biotech Lab, Department of Biological Sciences, Florida Atlantic University, DavieFL, USA

**Keywords:** plant invasion, soil microbial community, enemy release, biotic resistance, allelopathy, mycorrhizae, rhizobiome, mechanisms of invasion

## Abstract

Invasive plant species constitute a major ecological and economic problem worldwide, often distorting trophic levels and ecosystem balance. Numerous studies implicate factors ranging from environmental plasticity, competition for nutrient and space, and allelopathy in the success of invasive species in general. The Brazilian Pepper tree (BP) was introduced to the United States in the 1800s and has since become a category one invasive plant in Florida. It has aggressively spread to about 3000 km^2^ of terrestrial surface, fueled in part by the prevalence of the hybrid genotypes and environmental perturbations. It displays some of the well-established invasive mechanisms but there is a serious dearth of knowledge on the plant–microbe–soil interactions and whether the rhizobiome plays any roles in the displacement of native flora and the range expansion of BP. Several control measures, including chemical, mechanical, and biological antagonism have been used with limited success while restoration of natives in soils from which BP was removed has proved problematic partly due to a poorly understood phenomenon described as the “BP legacy effect.” Emerging evidence suggests that allelopathy, selective recruitment of beneficial soil microbes, disruption of microbial community structure and alteration of nutrient cycling, exhibited by many other invasive plant species may also be involved in the case of BP. This brief review discusses the well-established BP invasion mechanisms and highlights the current understanding of the molecular, below-ground processes. It also points out the gaps in studies on the potential role of microbial interactions in the success of BP invasion. These hitherto poorly studied mechanisms could further explain the aggressive spread of BP and could potentially contribute significantly to effective control measures and enable appropriate strategies for restoring native plants. The review advocates for the use of cutting-edge techniques in advancing the plant microbiome science. Ultimately, comparing metagenomic analyses of the rhizobiome of invasive plants grown in native and non-native soils could lead to a better understanding of the microbial determinants of biotic resistance, potentially empowering environmental managers with some predictive power of future trends of plant invasion.

## Introduction

Plant invasion is a global problem driven by human-mediated dispersal of plant species into new regions where they eventually acclimate, naturalize, and disturb populations of native plant species and multi-trophic ecosystems with consequent economic damage (Cuda et al., 2006). The term – ‘invasive plants’ refers to introduced plant species which establish, proliferate, and displace native flora, adversely affecting the habitat and ecosystem. Extensive global travels and international trading in the new global era contribute to the spread of invasive plant species across the world (Seebens et al., 2015). The impact of plant invasion is quite high. For example, the US Department of Interior estimate the total costs associated with invasive plants in general to be a whopping 123 billion US dollars per year (Cuda et al., 2006). These figures may even have been higher due to un-reported losses or costs for control and restoration but clearly illustrate the growing economic impact of invasion. More worrisome are those invaluable and perhaps irreplaceable losses of bio-resources and bio-diversity.

**FIGURE 1:**
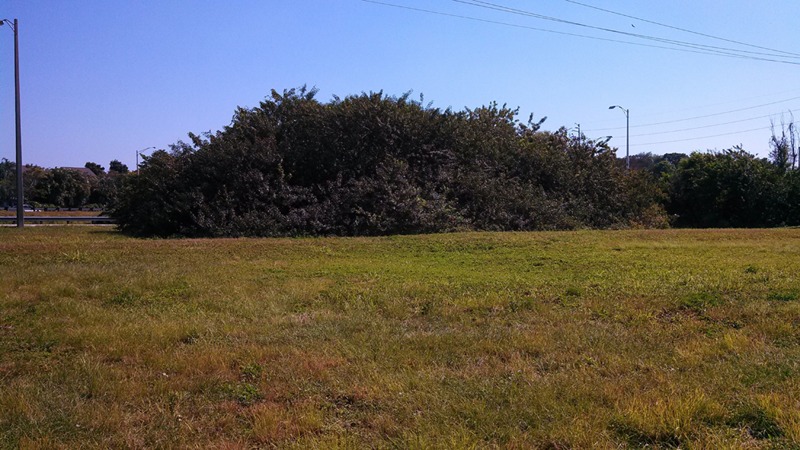
**Brazilian pepper tree (*Schinus terebinthifolius*) dominant stand in Broward County, Florida**.

**FIGURE 2:**
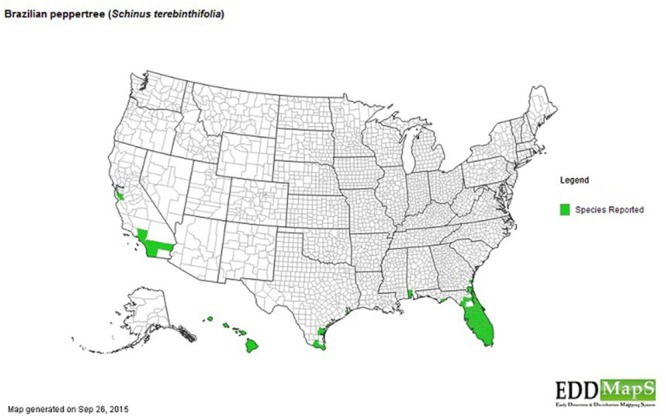
**Distribution of Brazilian pepper tree across USA and adjourning territories.** [Affected areas = Hawaii, California, Texas, Alabama, Georgia, and Florida shown in green (EDDMaps, 2015)].

The invasion of the Brazilian Pepper tree like other species is a multistage process including introduction (transportation), colonization, establishment, and range expansion (Theoharides and Dukes, 2007). The first step of the successful invasion of the Brazilian Pepper tree (**Figure 1**), a member of the family *Anacardiaceae* (cashew) began in 1898 when it was introduced to the USA from South America (Cuda et al., 2006). Some reports date its introduction back to 1832 (Mack, 1991) as an ornamental plant whose bright red fruits during the winter season were highly desirable, earning it the nickname, “Christmas berry” in Hawaii and the “Florida holly” in Florida (Cuda et al., 2006). Two haplotypes of the Brazilian Pepper tree (A and B) were introduced from two separate regions of Brazil into Florida where they became hybridized (Williams et al., 2005). The colonization stage of the BP in Florida involves surviving the relatively low threshold of prevailing abiotic filters such as sub-tropical warm temperatures and water stress. This has been significantly facilitated by its hybrid forms which grow more aggressively than their haplotype counterparts. The Brazilian Pepper tree has gained a foothold in Florida, Hawaii, Texas, and California (Cuda et al., 2006) with Florida and Hawaii being the most extensively colonized (Cox, 1999; Hight et al., 2003) as seen in **Figure 2**. Florida, Hawaii, and other tropical or sub-tropical regions are more susceptible to invasive plant species due in part to the abundance of disturbed environments and susceptibility of native flora (Lodge, 1993; Maron and Vila, 2001). Successful establishment of this category 1 invasive plant could be explained by high fecundity (Cuda et al., 2006), association with arbuscular mycorrhiza (Carneiro et al., 1996) and its physiologic adaptation to a wide variety of physico-chemical parameters such as pH, hydrology, and salinity. In addition, the low biotic resistance of Florida soils has been implicated in its susceptibility to invasion by exotic plants in general (Maron and Vila, 2001). There are no reports in literature about the precise role of soil microbiota in the establishment stage of the Brazilian Pepper tree invasion, even though the phenomenon is well known for other invasive plants such as numerous *Pinus* spp. (Richardson et al., 1994). It is conceivable that several below-ground interactions aid the adaptation of the BP to its non-native region of the world. The final step during plant invasion is range expansion. For the BP, which has caused remarkable ecological imbalance in a wide range of environments including disturbed sites, mangroves, pinelands, and hardwood hammocks (Ewel et al., 1982; Donnelly et al., 2008); anthropogenic factors coupled with high propagule dispersal rate and habitat connectivity are implicated. Rodgers et al. (2012) estimated that the Brazilian pepper tree is responsible for the loss of up to 2830 km^2^ of land mainly in Central and Southern Florida with an estimated 3000 km^2^ of terrestrial ecosystems affected. BP has been reported throughout all the islands of Hawaii and since 1998 was listed as one of the most significant invasive species affecting the general ecosystem (Hight et al., 2003). In Southern California, however, BP hasn’t been very successful but its very close relative *Schinus molle* (Peruvian pepper) has become naturalized (Nilsen and Muller, 1980). BP affects a multi-trophic system in the Florida Panther National Park where it displaced native plants, a food source for the white tailed deer whose population declined eventually impacting the food chain downstream (Maffei, 1997). More recently, BP has begun expanding its range beyond Florida, moving northward to Alabama, a relatively colder region (Mukherjee et al., 2012) which due to the rise in global temperatures accommodates plants that normally prefer warmer subtropical climates, eventually affecting plant diversity. Indeed, Duell et al. (2016) suggest that the future of plant invasion in grasslands worldwide will continue to be problematic as projected by new climate change models and the link between climate change and reduction in biodiversity is well known (Bakkenes et al., 2002; Keith et al., 2008)

In general, the success of plant invasion is determined by several interacting abiotic and biotic factors which define the susceptibility of the invaded habitat (Lodge, 1993; Maron and Vila, 2001), and the predisposition/traits of the invasive plant (Jose, 2002; Callaway et al., 2004; Batten et al., 2006). The susceptibility of the invaded habitat is also determined by the interactions of the invasive plant with soil microbes (Vitousek and Walker, 1989; Bains et al., 2009), native plants directly, and possibly a combination of the two. During colonization and enduring occupation of a given locality, invasive plants affect ecosystems by outgrowing native plants, modifying nutrient composition, soil carbon flow and microbial community structure of the soil among a myriad of other drivers. Parepa et al. (2013) noted that one of the major factors influencing the spread of invasive species is increased nutrient variability which interacts with other changes in environmental variability to substantially accelerate ecological change. While several of these factors have been implicated in other invasive species, there is a dearth of information on how interactions with soil microorganisms for example, specifically impact the successful invasion of the BP and the limited success of control measures.

In Florida for example, chemical control of the BP involves the use of herbicides such as triclopyr and imazapyr (Langeland and Stocker, 2001) which apart from being ineffective in dense stands of the BP may inhibit nearby plant flora (Laroche and Baker, 1994). Other control measures have been attempted without a practical and successful outcome. After a pilot test conducted by the state of Florida to eradicate BP from a 0.244 km^2^ plot in the Everglades National Park called the ‘Hole in the Donut’ by physically removing the plants using bulldozers, burning the plants and removal of the top soil, it was concluded that approximately US $20 million would be needed to restore 20 km^2^ with this method (Ferriter, 1997; Cuda et al., 2006); making mechanical intervention inefficient and impractical. Biological control measures included exploratory trials employing different arthropod species to reduce plant viability. No significant damage to BP plants was achieved by this method. A drupe feeding wasp damaged up to 31% of BP drupes during its main fruiting period (Wheeler et al., 2001) while the use of a fungal bio herbicide *Chondrostereum purpureum* which inhibited resprouting (Charudattan, 1996) proved minimally successful. The potential, however, exists for the fungus *Neofusicum batangarum* isolated from BP which was effective against seed germination and seedling growth of BP without inhibiting two other non-invasive plant species in Florida (Shetty et al., 2011). The native wax myrtle (*Myrica cerifera*) which is inhibitory to BP germination and seedling establishment employed by Dunevitz and Ewel (1981) and more recently by Overholt et al. (2012) did not provide significant success in controlling the BP in field studies. Meanwhile, bio-herbicides have been used extensively against some stubborn invasive weeds and have included formulations of different pathogenic bacteria and fungi such as *Pseudomonas* spp., *Xanthomonas campestris*, and *Colletotrichum gloeosporioides* among others. Given the almost non-existent data on the rhizobiomes of the BP, extending the promise of bio-control to the BP situation would require a careful study of its interactions with, and susceptibility to highly selective agents.

**FIGURE 3:**
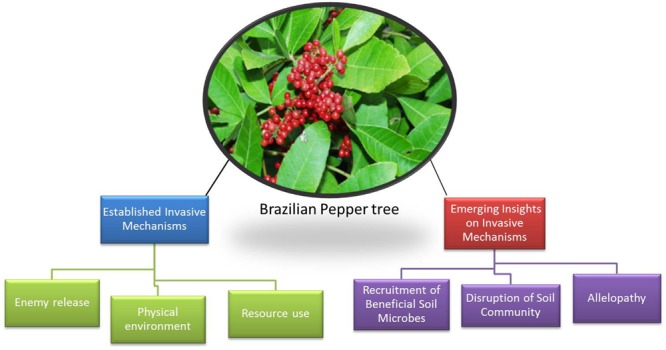
**Established and proposed emerging mechanisms of Brazilian pepper tree invasion in Florida and USA**.

To bolster ongoing efforts to control the BP and improve upon restoration of natives in these disturbed niches, it is very important that the complete picture of its mechanisms of invasion be deciphered. In this review, we discuss the established or well-studied plant mechanisms of invasion in the BP and highlight key emerging mechanisms and research gaps in (a) the current understanding of the molecular, below-ground processes underlying known BP invasion processes and (b) studies on the potential role of microbial interactions in the success of BP invasion which are otherwise established for other invasive species. These hitherto poorly studied mechanisms could further explain the aggressive spread of the BP. Such knowledge would contribute significantly to development of effective and sustainable control measures and enable appropriate strategies for restoring native plants. Several studies have shown that BP displays the well-known plant mechanisms of invasion such as competitive resource use, enemy release and physical environment mechanisms (**Figure 3**) but there is a serious dearth of knowledge on the plant–microbe–soil interactions and whether the rhizobiome plays any direct roles in the displacement of native flora and the aggressive growth of the invading species. Moreover, it is becoming increasingly clear that the molecular basis of known plant mechanisms of invasion involve soil microorganisms and their metabolites.

## Plant Invasion Mechanisms of Brazilian Pepper Tree

In general, plant mechanisms of invasion involve several complex processes which have been addressed by various reports (Levine et al., 2003; Rai, 2013, 2015). One of the cross-cutting frameworks by Ren and Zhang (2009) categorize these mechanisms into three major hypotheses namely the Adaptation to physical environment, Resource use and the Enemy release hypothesis. Many reports (Carneiro et al., 1996; Ewe and Sternberg, 2002; Morgan and Overholt, 2005; Spector and Putz, 2006; Geiger et al., 2011; Mukherjee et al., 2012) have linked these general mechanisms to the case of BP where its high fecundity, ease of environmental adaptations and increased competitiveness in its non-native region exemplify these classic plant mechanisms of invasion. There is, however, a growing understanding that the underlying processes of invasion are complex with overlapping spatial and temporal manifestations (Callaway and Ridenour, 2004; Blumenthal et al., 2009; Rai, 2013). The three interconnected mechanisms (physical environment, resource use, enemy release) reported for BP can also the termed the “niche opportunity hypothesis” which include the environmental variations through space and time (Rai, 2013). The niche opportunity describes the conditions that allow an invasive plant to take advantage of its “natural enemy release,” in a nutrient rich non-native environment by quickly adapting and eventually out-competing the native plants. An extension of this natural enemy release includes the “novel weapons hypothesis” (Callaway and Ridenour, 2004; Rai, 2013) where invasive plants exert greater allelopathic effects in the non-native region where the native plants and soil biota have not co-evolved. The “evolution of increased competitive ability (EICA)” (Callaway and Ridenour, 2004; Rai, 2013) also coincides with the enemy release hypothesis. In this scenario, the invasive plant which escapes from its natural enemies will no longer need the energy expended on defense strategies which will then be transferred to the enhancement of its competitive and reproductive abilities, seen in the below sections for the BP. The novel weapons hypothesis is webbed with yet another mechanism referred to as “allelopathic advantage against resident species (AARS) hypothesis” where plants in non-native regions evolve greater concentrations of allelopathic chemicals compared to their native region, providing the invasive plant with a competitive advantage. Details of these newer hypotheses have not been demonstrated for the BP. The enemy release hypothesis and all its extensions readily explains why some invasive plants are not as effective in their native regions which is quite the case for BP where its native form is a less aggressive (Geiger et al., 2011) colonizer. Many of these combined and extended mechanisms are exhibited during the four stages of BP plant invasion, being dependent on propagule pressure, characteristics of the invading species and susceptibility of the new environment including natural and anthropogenic disturbance (Rai, 2013). The already established mechanisms of BP explain how it successfully colonizes and spreads but little is known about the mechanisms it employs during establishment. To properly establish, invasive plants must overcome the biotic resistance (Levine et al., 2004) in the non-native region where plant–plant and plant–microbe interactions can influence or deter invasion. In fact little or nothing is known about the processes that lead to the establishment of BP invasion where the recruitment and alteration of soil microbiota potentially occurs and how the interactions are influenced by biotic resistance.

### Established Plant Mechanisms of Invasion in the Brazilian Pepper Tree

#### Physical Environment Mechanisms of the Brazilian Pepper Tree

During its colonization and range expansion stage, BP exhibits different adaptation to the physical environment. The hybrid form of BP found in Florida displays higher growth and survival rates than the native BP species in South America (Geiger et al., 2011). Although hybrid vigor may increase genetic variation and improve the potential for adaptation in non-native habitats, the release of BP from its natural enemies also contributes to its competitive and reproductive success. The absence of parasites and predators improve its viability and growth as described in the enemy release and evolution of increased competitive ability hypotheses. BP also has the ability to re-sprout vigorously from the main stems, and has a wide variety of insect pollinators. Humans are primarily responsible for seed dispersal through agricultural and industrial practices involving disturbance and movement of soil which contain many viable seeds in its non-native regions. The hybrid form of BP has considerable environmental tolerance to extreme moisture and salinity. Ewe and Sternberg (2002) showed that BP was tolerant to saline conditions and had similar salinity (sodium/potassium) ratios to mangroves. While this explains in part why it is able to thrive in the Everglades and invade the native mangrove areas of south Florida, the factors responsible for BP’s versatility and tolerance to high osmotic pressure and sodium toxicity are often not emphasized. In addition to being tolerant to salinity, BP also has the capacity to grow in shaded areas and can survive under elevated soil pH conditions. It is not clear, however, if the tolerance to pH, high osmotic pressure, and sodium toxicity is exhibited in its native range but BP seems to be able to survive in varied environments, from mangroves to pine hammocks. BP is dioecious with male and female flowers on separate plants which when pollinated creates increased genetic variation and the potential for adaptation to different environments. This adaptation has led to a phenotype plasticity observed by Spector and Putz (2006) where BP had the ability to change its growth form from a standing tree in monoculture plots to growing as a woody vine in invaded areas. Growing as a woody vine allows BP to cover a larger surface area and smother native plants. Using ecological niche modeling, Mukherjee et al. (2012) also showed that there is additional evolutionary adaptation to cold temperatures as BP spreads northward into Alabama and possibly further north due to climate change. Hybridization is considered a major factor in BP’s invasive success (Geiger et al., 2011) because increased genetic variation allows it to adapt well to the non-native environment. This genetic variability confers similar advantages as the enemy release hypothesis since release from natural enemies also provides competitive and reproductive advantages. These competitive advantages involve enhanced resource use efficiency.

#### Resource Use Mechanisms of the Brazilian Pepper Tree

Resource use mechanisms of BP are intertwined with the enemy release hypothesis where the effects of enemy release will be greatest for high resource use species that possess high nutrient uptake ability in resource rich environments (Blumenthal et al., 2009; Rai, 2015). The energy devoted to defense strategies against its natural enemies will offer a trade-off in energy supplied to enhancing competitiveness and fecundity in the non-native region. As a high resource use plant, BP has superior nutrient uptake ability compared to the native species, due to an extensive root system and strong association with arbuscular mycorrhiza (Carneiro et al., 1996). The plant also grows aggressively and laterally forming thick foliage which successfully competes with other plants for access to sunlight. Aeration caused by soil disturbances has been advantageous for BP as these soils are more favorable to mycorrhizal activity (Carneiro et al., 1996). This is one of the main reasons for the success of BP colonization in sites where there has been anthropogenic disturbance through construction or road development. BP was shown to have higher photosynthetic nitrogen-use efficiency than other native plants (Ewe and Sternberg, 2002), leading to superior growth and sustenance. Studies with other invasive exotics showed that they exhibited an increase in extractable nitrogen during invasions and created their own nutrient rich environment to promote growth (Ehrenfeld, 2003) which could be a possible case for BP. Li and Norland (2001) showed that there was a high association with the phosphorous content of BP and the available phosphorous levels in soils, indicating that soils high in phosphorous/phosphates may influence the invasion of BP. This identifies with a very common phenomenon where the availability of nutrients and water can enhance plant invasion (Rai, 2013). The availability of high phosphorous/phosphates concentrations in soil as a suitable indicator of potential invasion by BP has yet to be explored and would require an understanding of the soil microbial community’s structure and function.

#### Enemy Release Mechanisms of the Brazilian Pepper Tree

In invaded areas natural enemies such as seed predators, herbivores, plant pathogens, and competitors which are absent would allow proliferation of the invasive alien plant, normally controlled in the native region – a phenomenon coined as the enemy release or the natural enemy release hypothesis (Maron and Vila, 2001; Keane and Crawley, 2002; Levine et al., 2004). In a similar vein, biotic resistance hypothesis describes the ability of native plants or their associated soil microbes to constrain the effect of exotic invasion (Levine et al., 2004). An extension of the enemy release mechanism includes the evolution of increased competitive ability, which is a possible mechanism for BP. BP has been shown to out-compete native plants in a greenhouse study (Nickerson and Flory, 2014) and also out-performed the closest representation of the BP native plant (eastern and western haplotypes) introduced to Florida prior to hybridization (Geiger et al., 2011). A direct comparison with BP plants in its native and non-native region which would confirm this mechanism has not yet been undertaken. Studies, however, have shown that in BP’s native regions in South America, there are many natural enemies which presumably help to restrict its growth and reproduction. These natural enemies include a leaf fungus, various phytophagous insects, leaf feeding moths and weevils (McKay et al., 2009) but no in depth study has looked at the possible biotic resistance in native soils. A general theory was coined by Klironomos (2002) who stated that non-native invasive species escape host-specific pathogens in their native lands but connect with not host-specific soil mutualists such as arbuscular mycorrhiza in an invaded habitat. Although all these mechanisms have been implicated in BP invasion, there is still a huge gap in research and our understanding of exactly what defines the specificity of the natural enemies to the exotic plant in its native land. It is still not clear whether it is the absence of biotic resistance in the new region or the interaction of the invasive plant with the resident communities, including soil microorganisms that promote invasion possibly through allelopathic effects. In order to fully delineate the effects of the enemy release hypothesis, studies should include a comparison of the microbial community structure in rhizospheric soil from the native and non-native habitats, in the presence and absence of the target invasive plant. The generally low biotic resistance of Florida soils (Maron and Vila, 2001) is conducive to plant invasion for which BP has gained a competitive advantage boosted by its other established invasive mechanisms of invasion. These three established inter-connected mechanisms of BP which encompasses the niche opportunity hypothesis (physical environment, resource use, and enemy release) are manifest in the introduction, colonization, and range expansion stages of the BP invasion but other below-ground mechanisms which potentially drive the establishment stage are poorly defined.

### Emerging Insights into the Plant Mechanisms of Invasion in the Brazilian Pepper Tree and the Link with Other Invasive Species

#### Allelopathy and the Possible Role of Soil Microbes

Allelopathy is the relationship between two or more organisms, including plants where one thrives by actively inhibiting the other(s) by producing targeted biochemical compounds (Cipollini et al., 2012). Allelopathy is also described and involved in the novel weapons hypothesis as a scenario where the native community is not adapted to the biochemical compounds produced by the invader (Hierro and Callaway, 2003; Callaway and Ridenour, 2004). The AARS occurs where invasives evolve greater concentrations of allelochemicals in their non-native than their native range (Rai, 2013). This could provide the BP plant with a competitive advantage over the native plants.

Allelopathy is still being debated among plant ecologists as an important invasive invasion and most recently in the case of BP, it was denounced as a plant mechanism of invasion by Nickerson and Flory (2014). They had surmised that competition, not allelopathy was the cause of biomass reduction seen in Florida natives tested in greenhouse experiments with and without activated charcoal which adsorbs allelochemicals. The authors, however, did not explore the changes in the microbial communities that may have occurred during their experiments, leaving a void in understanding the role that the soil microbial community may have played in the reduced biomass of the native plants. No study so far has elucidated in depth, the rhizospheric microbial flora of BP or the systematic community composition and structural changes that occur during invasion. Fabbro and Prati (2015), also did experiments which explored another angle where not only the effects of invasive species with natives were studied, but also native with native plant. They concluded that native plants may also induce negative effects on growth of other native plants. They discovered by testing multiple native and invasive plants that there was no significant difference between the negative effects on growth induced by an invasive plant and those induced by natives themselves. Their sterile experiments also showed that allelopathy was not inhibiting the growth of the native plants. The nature of allelopathy indicates that it tends to be more pronounced where exotic species gain access to new, native environments which do not share an evolutionary history with the invasive plants or the allelochemicals they produce (Keane and Crawley, 2002). An invasive species’ native environment may possess an arsenal of evolutionarily inclined microbes that could counter or neutralize the effects of the plant-produced allelochemicals through degradation (Levine et al., 2004) or due to the lack of evolutionary interaction in the non-native region unable to degrade the allelochemical to prevent invasion. In one study by Willis (2007), it was suggested that allelochemicals rarely reach toxic levels in the soil due to microbial degradation. Another study by Lankau (2010) found that allelopathic inhibition of the sycamore tree (*Platanus occidentalis*) by garlic mustard (*Alliaria petiolata*) was influenced by the soil microbiota present; the inhibition was observed only in sterile soils, suggesting that the soil microbes may have degraded the allelochemicals produced. Lankau (2010) and Fabbro and Prati (2015) have shown that studying the role of microbes in allelopathy requires conducting a sterile vs. non-sterile soil experiment. The sterile soil will definitely show if the soil microbes present influence or inhibit the effect of any allelochemical produced and a similar study has to be done with BP to evaluate these effects.

It was discovered earlier by Callaway et al. (2008) that the allelochemicals produced by garlic mustard (*A. petiolata*) were glucosinates which altered the composition of arbuscular mycorrhiza. This invasive plant more recently was shown to inhibit arbuscular mycorrhizal (AMF) and ectomycorrhizal (EM) mutualists needed by native plants (Callaway et al., 2008). The use of glucosinates by *A. petiolata* which inhibits the growth of fungal mutualists provides the biochemical basis of its allelopathic effect and is an extension of the AARS (Rai, 2015). It is also known that some plants may form symbiotic relationships with specific microbial populations in their rhizosphere which could enhance the effects of the allelochemicals the plant produces, influencing how conspecific and heterospecific individuals respond while growing in the same soil (Cipollini et al., 2012). A black walnut tree (*Juglans nigra*) was reported to release the allelopathic compound ‘juglone’- a plant respiratory inhibitor in the soil in large measurable quantities. This allelochemical is believed to be the cause of the absence of other species of plants normally seen in close vicinity to the tree (Jose, 2002; Cipollini et al., 2012). Dense monocultures of BP with no natives in close proximity are not uncommon, and have been largely attributed to its height and density which obstruct sunlight from reaching other plants. Is it possible that there are yet-to-be defined allelopathy phenomena exerted by BP? Potential allelopathic effects could stem from its leaves and seeds which possess natural essential oils and antimicrobial compounds such as alpha-pinene and limonene (Singh et al., 1998). The extract from BP bark has also been found to have genotoxic effects by causing damage and mutations in bacterial DNA (de Carvalho et al., 2003). Its use as an herbal medicine for human ailments and bacterial and fungal infection (Cuda et al., 2006) also suggest its antimicrobial activity. BP extracts were found to prevent the growth of gram positive bacteria and pathogenic fungi at the clinical level (Alves et al., 2013; Gomes et al., 2013) and specifically inhibit the cell wall proliferation in certain *Candida* spp. (Johann et al., 2010). Aqueous extracts of BP leaves were shown to inhibit seed germination and growth of two Florida native plants: shepherd’s needles (*Bidens alba*) and pigeon berry (*Rivina humilis*; Morgan and Overholt, 2005). BP seeds actively reduced the growth of red and black mangrove seedlings from the Florida Everglades (Donnelly et al., 2008). It is quite remarkable that none of the published studies have demonstrated the allelopathic effects of BP on natives or soil microbiota in the field. BP’s potential as an allelopathic plant could be linked to the presence of phenolic acid compounds in water soluble extracts of its seeds (Nilsen and Muller, 1980). BP is known to produce a variety of terpenes and phenolic compounds (Cuda et al., 2006) and phenols and sugar alcohols are the main chemicals used by plants to alter soil microbiota (Badri et al., 2013). Is it possible that BP could be using these chemicals to recruit beneficial microbes and suppress others needed by native plants? It is still unclear how these compounds produced by BP affect native plants and the microbiota in the field therefore more work needs to be directed at this level of study. In all, it cannot be ruled out yet that BP exhibits allelopathic effects on its environment to achieve its invasiveness, although the chemical or molecular mechanisms of the allelopathy by BP or associated soil microbes (if it exists) are still not clear. To further investigate this phenomenon, a metabolomics study could be conducted with sterile and non-sterile soil samples where additional analysis on culture and metagenomics data is done temporally as the BP plant grows with and without native plants. This will show the microbial community dynamics and metabolic changes that occur overtime which should provide better clues of its plant mechanism of invasion. The soil microbiome may have positive and negative effects toward competing plant species. Two arguments raised here could be that allelochemicals produced by the plant may be enhanced by certain microbes in the soil by converting them into more toxic by-products which has not been fully studied, or that some soil microbes somehow do not affect the degradation or modification of certain allelochemicals (Bakker et al., 2013). A combination of the established plant mechanisms of invasion in BP along with an emerging plant mechanism of invasion such as allelopathy with the novel weapons hypothesis could explain the legacy effect reported by Nickerson and Flory (2014) who described the observation that after BP plants were mechanically uprooted other Florida native plants find it difficult to grow. If the half-life of a putative allelochemical is high; then the explanation of the observed scenario is not far-fetched. However, other studies have shown that allelochemicals usually have a short half-life due to degradative processes that may occur in soil (Willis, 2007; Cipollini et al., 2012), suggesting that the ‘legacy effect’ of BP could be related to a more stable alteration of nutrient cycling and microbial community structure of detrimental consequence to native plants. Additionally, Cipollini et al. (2012) discussed the long term effect of leaf and seed litter which may also boost the legacy effect theory as the seeds, leaves, roots, and stems have been shown to inhibit seed germination and possess antimicrobial attributes. In another interesting study by Barto et al. (2011) it was shown that allelopathy can be enhanced/spread by arbuscular mycorrhiza in the local non-native region. BP is known to associate with the common AMF in soil and could also employ them to distribute its allelochemicals farther than the reach of the roots. Pringle et al. (2009) mentioned that AMF may be transported with an invasive plant increasing their spread in a non-native range. In the case of BP, seeds and leaves consisting of alellochemicals or associated microbes are easily distributed and can influence the soil microbiota and inhibit native plant growth in a new non-native range. This BP ‘legacy effect’ brought about by allelopathy and the possible interaction of soil microbes does explain the ability of this plant to colonize and establish dominant local stands wherever one plant invaded. The soil environment it creates is conducive to its growth while limiting the success of other natives. Studies which include the systematic monitoring of changes in allelochemical concentrations, nutrient enrichment or depletion and soil microbial community structure would be able to differentiate the specific legacy effect of BP. Control of invasion as well as restoration efforts would then have to include neutralization of allelochemicals deposited in the soil to restore the disrupted soil community and cure the BP legacy left in the soil. A summary of different allelochemical-mediated mechanisms of selected invasive plants compared with present research on BP is shown in **Table 1**.

**Table 1:** Selected allelochemical and microbe mediated mechanisms of plant invasion exhibited by well-studied invasive plant species compared to the Brazilian Pepper tree status quo.

Alellochemicals/Soil microbe(s)	Invasive plant involved	Method of action	Brazilian Pepper tree Status quo	Reference
Juglone	Black walnut (*Juglans nigra*) Secrets Juglone^∗^	Juglone – selectively inhibits respiration of nearby plants	No known direct allelochemical discovered. Plant extracts inhibit seed germination in native plants^∗∗^	Jose, 2002^∗^; Morgan and Overholt, 2005^∗∗^; Donnelly et al., 2008
Sodium ions	Salt lover (*Halogeton glomeratus*) Extrudes sodium ion to the environment^∗^	Alteration of soil microbial and plant communities via increased sodium toxicity	No known or similar mechanism discovered. High phosphate concentrations are associated with BP invaded soils^∗∗^	Li and Norland, 2001^∗∗^; Duda et al., 2003^∗^
8-hydroxy-quinolone	Diffuse knapweed (*Centaurea diffusa*) Root microbiota benefits plant and secrets antimicrobial^∗^	Alteration of soil microbial composition via 8-hydroxy–quinolone antibacterial effects	Numerous anti-microbial compounds recovered from BP^∗∗^. Links to plant invasion are vague.	Singh et al., 1998; Callaway et al., 2004^∗^; Gomes et al., 2013^∗∗^
Glucosinates	Garlic mustard (*Alliara petiolata*) Roots produce glucosinates^∗^	Alteration of composition of arbuscular mycorrhiza (AM) in soil	No glucosinates recovered from BP yet and no reports on similar mode of action despite clear AM involvement	Callaway et al., 2008^∗^
*Frankia* spp.	Firetree (*Myrica faya*) Recruits nitrogen-fixing bacteria - *Frankia* spp. ^∗^	Colonize nitrogen-limited soils, altering plant community structure	Similar studies are scarce for BP. Recruitment of such soil microbial species unknown	Vitousek and Walker, 1989^∗^
Mycorrhizal fungi	Pine (*Pinus* spp.) Recruit mycorrhizal fungi^∗^	Superior resource use mechanism. Improves growth and colonization	Known to recruit Mycorrhizal fungi^∗∗^. Exhibits efficient resource use mechanisms and nutrient uptake	Richardson et al., 1994^∗^, Aziz et al., 1995^∗∗^
Sulfur oxidizing and sulfur reducing bacteria, arbuscular mycorrhizae	Yellow starthistle (*Centaurea solstitialis*) Recruits beneficial soil organisms^∗^	Competitive advantage with altered rhizosphere microbiota composition	No such studies have tied sulfur oxidizing or reducing bacteria in BP invasion	Batten et al., 2006^∗^
Sulfur oxidizing and sulfur reducing bacteria, arbuscular mycorrhiza	Barb goatgrass (*Aegilops triuncialis*) Recruits beneficial soil organisms^∗^	Competitive advantage with microbial association and altered rhizosphere microbiota composition	No such studies have tied sulfur oxidizing or reducing bacteria in BP invasion	Batten et al., 2006^∗^
*Glomus geosporum*	Forb (*Solidago canadensis*) Specifically associates with *G. geosporum* and suppresses the prevalence of a widespread AM – *G. mosseae*^∗^	Disruption of soil mycorrhizal community to the detriment of natives.	BP has been shown to recruit *G. geosporum* AM in soil^∗∗^ but no studies have demonstrated the detrimental effect on natives if any.	Aziz et al., 1995^∗∗^; Zhang et al., 2010^∗^
*Rhizobium* spp. and *Azotobacter* spp.	*Polygonum avuncular.* Inhibits proliferation of *Rhizobium* spp. and *Azotobacter* spp.^∗^	Indirect Allelopathy via reduction of n-fixing rhizobacteria and *Azotobacter* populations	No such studies have tied the reduction of beneficial rhizobacteria and *Azotobacter* by BP during invasion	Alsaadawi and Rice, 1982^∗^

For each row, *well studied mechanisms of other plants and corresponding reference. **Case of BP and the corresponding reference.

#### Selective Recruitment of Beneficial Soil Microbes by Brazilian Pepper Tree and Other Invasive Plants

Plants generally dictate the types of rhizosphere microbes they recruit which are heavily influenced through the production of plant exudates, such as flavonoids and other hormone products. Specifically, the different genotypes of plants determine whether the microbiome community serves a beneficial or pathogenic role (Haney et al., 2015) where they are actively involved in the construction of parts of their own microbiome (Stone, 2016). Even plants within a single species have varied rhizobiome communities observed in a study done on *Arabidopsis* genetic variants (Haney et al., 2015). They also showed that the host genotype has a minor but significant effect on the rhizosphere community. The hybrid form of BP in Florida has produced a plant with increased vigor and reproductive capabilities, but this may create another factor of invasion by microbe mediated adaptation through hybridization. It is hypothesized that the hybrid BP has evolved new mechanisms to recruit beneficial rhizosphere microbes while excluding pathogens which has aided its establishment.

Mycorrhizal fungi form one of the strongest mutualistic relationships with plants because they have the ability to improve the availability of nutrients to plants including phosphates, increase water uptake and reduce abiotic and biotic stress (Jansa et al., 2013). AMF are quite common in tropical soils and are not known to be particularly host specific (Klironomos, 2002) but have been more recently shown to associate with particular plant functional groups (Lekberg et al., 2013). Generalist plant invaders tend to be more successful invaders as they form symbiosis with more common soil micro-organisms compared to specialized invaders that rely on certain microbes that may not be present in different geographical locations and may not spread as wide (Pringle et al., 2009). Numerous *Pinus* spp., invasive plants in the Southern hemisphere was shown by Richardson et al. (1994) to be associated with common mycorrhizal fungi during invasion which benefit the plants as the association allows them to acquire more nutrients and out-grow native species. In areas devoid of these symbionts, the *Pinus* spp. was unable to establish itself fully. Aziz et al. (1995) showed that BP was associated with the most relative abundant AMF in soil including *Glomus geosporum* and *Glomus etunicatum*. This study gives the first indication of the specific AMF associated with BP and shows that it is considered as a generalist invader and gives plausible reasons for its quick establishment of dominant mono-culture stands during its invasion process. It is still unclear, however, why if BP recruits beneficial microorganisms in its invasive efforts how the natives are negatively affected. It is quite possible to speculate that again as shown by Barto et al. (2011), they could be using the wide fungal mycelial network to distribute allelochemicals to other neighboring native plants as discussed previously during colonization and establishment. These beneficial AMF microbes will provide the necessary nutrients required to enhance plant cover of the invasive species but serve a sinister role by negatively affecting native plants again creating the ‘BP legacy.’ Another study by Lekberg et al. (2013) showed that dominant monoculture stands of the mycotrophic invasive species knapweed (*Centaurea maculosa)* and leafy spurge (*Euphorbia esula*) showed an increase in AMF abundance and richness. It is still unclear, however, what specific AMF BP recruits and how these proposed mechanisms of microbial recruitment or suppression function. While it has been shown where the symbiosis with fungal mutualists exists there is paucity in research regarding the association of BP with rhizobacteria and any benefits or advantages that they may induce during plant invasion. Studies are needed to elucidate the molecular factors that regulate the complete rhizobiome community in a very complex ecological niche. This should also include a comparison of the relative abundance of fungal and bacterial micro-organisms which may denote a certain recruitment strategy at the Kingdom level exhibited by invasive and even non-invasive plants (Callaway et al., 2008; Turner et al., 2013). A summary of selected microbe-mediated mechanisms of invasion and the need for exploring similar mechanisms in BP are shown in **Table 1**.

#### Disruption of the Soil Microbial Community Structure by Brazilian Pepper Tree

Some invasive plants have been shown to cause soil bacterial community shifts mainly by reducing the abundance of particular beneficial microbes such as plant growth promoting (PGP) bacteria and AMF (Kourtev et al., 2002; Batten et al., 2006; Callaway et al., 2008; Inderjit and van der Putten, 2010) just to name a few. To disrupt the soil microbial community of the non-native range during establishment, the invasive plant has to first overcome the natural biotic resistance of the soil. Biotic resistance may have neutral to positive benefits for invasive plants while mostly being negative for native plants (Inderjit and van der Putten, 2010). Maron and Vila (2001) reported that Florida, soils have a high susceptibility or low biotic resistance to invasive plants and may be the main reason why Florida is inundated with different invasive species. No study has fully elucidated the effect BP has on the soil community structure during invasion or if there is any clear understanding of what constitutes “low biotic resistance” with respect to microbial community changes and it is imperative that these studies be undertaken. Most native plants thrive in the presence of AM fungi (Aziz et al., 1995) which have also been responsible for altering the soil community structure (Aziz et al., 1995) by unknown mechanisms. It was shown in California and Florida that native plants rely more on AMF than non-native plants (Vogelsang et al., 2005; Lin et al., 2011). Native plants showed an AMF growth response of 82% greater than that seen in non-native species (Vogelsang et al., 2005). A study by Zhang et al. (2010) further brought this invasion mechanism into perspective when they found that an invasive forb *Solidago canadensis* changed the AMF community mutualism normally found with a native forb species *Kummerowia striata*. The invasive plant increased the abundance of one AMF species (*Glomus geosporum*) to its benefit while decreasing the prevalence of a widespread AM fungus found in the soil (*Glomus mosseae*) to the detriment of the native species. The exact mechanism is unknown but this phenomenon fits the degraded mutualism hypothesis where invasive plants alter the soil microbiota by reducing the AMF abundance and richness (Lekberg et al., 2013). The symbiosis between AMF and plants through unknown mechanisms may also activate the expression of plant genes required for uptake of inorganic phosphorous, nitrogen and other nutrients from depleted soils. Lin et al. (2011) showed that the infection of sawgrass (*Cladium jamaicense*) in the Florida Everglades with AMF initiated the activation of a phosphate transfer gene which allowed the uptake of Pi from soil. It is quite plausible that invasive plants such as BP alter the soil microbial community much more significantly than is currently known. By recruiting or selecting for AMF and certain beneficial bacteria which facilitate nutrient cycling and pathogen protection, invasive plants gain a competitive advantage. Sulfur oxidizing, and sulfur reducing bacteria were found predominantly in the rhizosphere of two invasive species yellow starthistle (*Centaurea solstitialis*) and barb goatgrass (*Aegilops triuncalis*) while they were found in substantially less proportions in native soil rhizosphere (Batten et al., 2006). A similar pattern was observed earlier by Aziz et al. (1995) in dominant BP stands where AMF diversity remained unchanged but after soil removal and physical uprooting of BP, there was an increase in AMF activity in the soil which enabled the re-growth of different native species. This provides a clue of how the reduction of these beneficial microbes needed by native plants can drive invasion through local establishment of BP. BP has been shown to have a strong association with arbuscular mycorrhiza and it remains to be shown whether there is any selective association in invaded habitats. It is, however, evident that the presence or absence of key soil microorganisms manipulated by invasive plants does improve the establishment phase of their invasion (Richardson et al., 1994; Batten et al., 2006) and also directly impacts native plants in the same locality (Callaway et al., 2008; Zhang et al., 2010). The evidence of the propensity of BP to alter soil microbiota and inhibit seed germination is continuously mounting and will hopefully lead to a better understanding of the below ground plant mechanisms of invasion in BP. A complete analysis of various BP extracts show the potential for antimicrobial activity *in vitro* but it is yet to be seen how this plays out in the field. Studies are also wanting in the areas of assessing the direct effects of extracts of beneficial soil microbes on native plants in the field to counter any adverse effects resulting from shifts in soil microbial symbionts needed by the resident species. Environmental restorative efforts would be greatly enhanced by replenishing formerly invaded areas with the beneficial microbes that were displaced through microbial inoculant technology.

## The Need To Include Advanced Molecular Methods in the Analysis of the Soil Rhizosphere Microbiome

It has been shown so far that the manipulation of soil bacteria and fungi exerts profound impacts in plant invasion and should help in shedding some light on the plight of ecosystems invaded by BP. Culture-dependent techniques have been widely used in Microbiology to assess the morphological and physiological traits of different microbes but this only captures a small portion of all micro-organisms as millions are still un-cultivable or yet-to-be-cultured (YTBC). New nanotechnologies such as microfluidics hopefully will uncover these recluse ‘hermit-like’ microbes in their natural environment. Phospholipid fatty-acid analysis (PLFA) has also been widely used to determine the structural diversity of soil microbiota (Wolfe and Klironomos, 2005). This non-culture dependent method uses the changes in phospholipid production to resolve broad groups of microorganisms such as bacteria and fungi only down to the Genus level but has been beneficial in denoting soil microbial community changes during plant invasion (Kourtev et al., 2002). Nucleic acid analysis has moved to the forefront in analysis of soil microbiota community changes during plant invasion (Wolfe and Klironomos, 2005). Fingerprints of microbial communities were first assessed using denaturing gradient gel electrophoresis (DGGE) and terminal restriction length polymorphisms (t-RFLP). The most modern methods for studying shifts in soil microbiota communities include metagenomics studies where chronometer genes, not subject to horizontal gene exchange in bacteria and fungi are sequenced to analyze the microbiome and their functional dynamics (Jansson et al., 2012). High-throughput sequencing platforms such as 454 by Roche and the MiSeq/HiSeq by Illumina have allowed the enhanced resolution of microbiomes with millions of sequencing reads and should be adopted in plant invasion studies. In terms of food crops, detailed rhizosphere structure at the Order and Genus level has already been obtained for potato, rice, maize, and others food crops through metagenomics studies (Tkacz and Poole, 2015). Research done on the rhizosphere of the pea plant has shown that incredibly significant differences in the rhizosphere microbial community may be occurring at the domain or kingdom level where the pea plant supports a higher eukaryotic population compared to prokaryotes (Turner et al., 2013). These soil microbial diversity studies coupled with metabolomics bio-assays are the key to deciphering many obscure plant mechanisms of invasion which have not been studied in the Brazilian pepper tree’s invasion. Using RNA metatranscriptomics, the active microbiomes associated with the plant rhizosphere were deciphered (Turner et al., 2013) and could be the key to understanding the role of microbial (bacteria and/or fungi) genes in the plant mechanism of invasion in BP and other invasive plants. With these metagenomic studies it is possible to ascertain if BP is also recruiting its own beneficial soil microbes and altering the adjacent soil community to its own benefit and the demise of native plant species. If so, we may also elucidate the specific type of soil microbes they are normally associated with and from what kingdom. From this information knock-down and restoration strategies can be developed through the use of soil microbial inoculants to prevent establishment of BP and improve re-establishment of natives. Abiotic and biotic soil and rhizosphere analyses of invasive plants and previously invaded areas using these advanced techniques are promising. Analysis of the rhizobiome of invasive plants in their native and non-native region along with the microbial soil flora in each region should provide some predictive power for future plant invasions and hopefully prevent them. An un-invaded area devoid of soil microbial communities which can control the establishment of an invasive plant while being prevalent in microbial soil mutualists that they can manipulate may have the potential to be invaded. This was also involved in the well-studied invasive plants *C. maculosa* (Callaway et al., 2004) and *A. petiolata* (Callaway et al., 2008).

## Conclusion

The elimination/control of BP and indeed all exotic species, and the restoration of native plant communities continue to be a growing challenge and concern for ecologists. The problem will be compounded by predicted impact of climate change, underscoring the need for more research to fuel innovation in control measures. The BP plant, like other invasive species has many known advantages over native plants and possesses multiple established plant mechanisms of invasion including the traditional physical environment adaptation, high resource use efficiency and enemy release mechanisms (niche opportunity). These interconnected established mechanisms have been shown to enhance invasiveness during introduction, colonization and spread of BP. Mechanisms emerging as important employed by exotic species and possibly the BP include the use of allelochemicals to manipulate the soil community structure and the recruitment of AMF fungi for enhanced nutrient uptake, drought tolerance, pathogen resistance, and disruption of the native soil microbe community. These important emerging mechanisms shed some light into the dominance of BP during establishment of local populations. Whether soil microbes influence allelopathy, or are recruited by invasive plants during invasion, they seem to play a significant role. The soil microbial community under BP has still not been fully elucidated but new high resolution advanced molecular studies are needed to analyze in depth the bacterial and fungal soil community structural changes to determine their role in plant invasion. These advanced high throughput metagenomic studies should be integrated with metabolite/biochemical bio-assays and plant gene expression research to better understand plant mechanisms of invasion and the vulnerability of native species. This total rhizosphere community DNA can provide evidence of plant species’ specific microbiomes (Bakker et al., 2013; Tkacz and Poole, 2015) but it’s also essential that these studies involve a spatial and temporal aspect due to the different dynamics of soil microbiota based on geography and seasons, respectively. To even further confirm some of the established strategies such as the enemy release hypothesis, comparisons between native and non-native ranges should be conducted using these new methods. Importantly, the biotic resistance of Florida soils could be analyzed using a combination of metagenomics and functional genomic analyses where the abundance and richness of soil microbiota in a given geographical location could be used to establish models that allow environmental managers some predictive power of future trends of plant invasion. Knowledge from these emerging frontiers could lead to the development of new knockdown strategies by disruption of their soil microbiota or the restoration of invaded areas with beneficial soil microbial inoculants and the potential creation of predictive tools for planning and managing plant invasion.

## Author Contributions

KD contributed equally to the research, drafting and editing of the manuscript. NE is the senior author who guided the research, contributing to research, drafting and editing of manuscript.

## Conflict of Interest Statement

The authors declare that the research was conducted in the absence of any commercial or financial relationships that could be construed as a potential conflict of interest.
